# Nephronophthisis

**DOI:** 10.1007/s00467-008-0840-z

**Published:** 2008-07-08

**Authors:** Rémi Salomon, Sophie Saunier, Patrick Niaudet

**Affiliations:** 1 Pediatric Nephrology, Centre de référence des Maladies Rénales Héréditaires de l’Enfant et de l’Adulte (MARHEA), Hôpital Necker-Enfants Malades, Paris, France; 2 INSERM U 574, Hôpital Necker-Enfants Malades, Paris, France; 3 Service de Néphrologie Pédiatrique, Hôpital Necker-Enfants Malades, 75743 Paris Cedex 15, France

**Keywords:** Nephronophthisis, Cystic kidney disease, Chronic tubulointerstitial nephritis, Chronic renal failure, Senior-Løken syndrome, Ciliopathy

## Abstract

Nephronophthisis (NPH) is an autosomal recessive disease characterized by a chronic tubulointerstitial nephritis that progress to terminal renal failure during the second decade (juvenile form) or before the age of 5 years (infantile form). In the juvenile form, a urine concentration defect starts during the first decade, and a progressive deterioration of renal function is observed in the following years. Kidney size may be normal, but loss of corticomedullary differentiation is often observed, and cysts occur usually after patients have progressed to end-stage renal failure. Histologic lesions are characterized by tubular basement membrane anomalies, tubular atrophy, and interstitial fibrosis. The infantile form is characterized by cortical microcysts and progression to end-stage renal failure before 5 years of age. Some children present with extrarenal symptoms: retinitis pigmentosa (Senior-Løken syndrome), mental retardation, cerebellar ataxia, bone anomalies, or liver fibrosis. Positional cloning and candidate gene approaches led to the identification of eight causative genes (*NPHP1, 3, 4, 5, 6, 7, 8,* and *9*) responsible for the juvenile NPH and one gene *NPHP2* for the infantile form. NPH and associated disorders are considered as ciliopathies, as all *NPHP* gene products are expressed in the primary cilia, similarly to the polycystic kidney disease (PKD) proteins.

## Introduction

Nephronophthisis (NPH), an autosomal recessive disorder initially described in 1945 by Smith and Graham and in 1951 by Fanconi, is a chronic tubulointerstitial nephritis that uniformly progresses to end-stage renal disease (ESRD) [1, 2]. With regard to the age of onset for ESRD, three clinical variants have been described: infantile, juvenile, and adolescent forms [3]. Of these, juvenile NPH is the most common, which accounts for 5–10% of cases of ESRD in children. In the past, NPH and medullary cystic kidney disease (MCKD) were considered in the same complex. Whereas these disorders share a number of clinical as well as histological features (tubular basement membrane disintegration, tubular cyst formation, and tubulointerstitial inflammation and fibrosis) [4–6], MCKD is distinct from NPH by its autosomal dominant inheritance and by the late onset of renal failure after the fourth decade of life [7]. In this review, we only consider NPH.

### Juvenile nephronophthisis

Juvenile NPH is an uncommon condition that affects girls and boys equally. The incidence is approximately 0.13 for 10,000 live births in Finland, whereas in Canada, it is 1 per 50,000 live births and in United States 9 per 8.3 million [8–10]. The disorder has been reported worldwide. The first symptoms generally develop around 4–6 years of age. Polyuria and polydipsia related to a reduced urinary concentrating ability and loss of sodium conservation occurs early in the course of the disease, whereas glomerular filtration rate (GFR) remains normal [11]. Decreased urinary concentrating defect is demonstrated by a low urinary osmolarity (<400 mosm/kg in the first urine sample in the morning), which does not increase after desmopressin acetate administration [5]. Urinary sodium wasting may be responsible for hyponatremia and hypovolemia in cases of decreased sodium intake. Decreased growth velocity related to chronic dehydration and later to renal insufficiency results in growth retardation. Hematuria and proteinuria are absent or minimal. Blood pressure is normal before the onset of renal failure.

Renal insufficiency is often present when the diagnosis is made. Late symptoms are related to the progressive renal insufficiency and include anemia, metabolic acidosis, nausea, anorexia, and weakness. ESRD develops at a mean age of about 13 years but can also occurs in some rare cases much later during adulthood [12, 13].

**Fig. 1:**
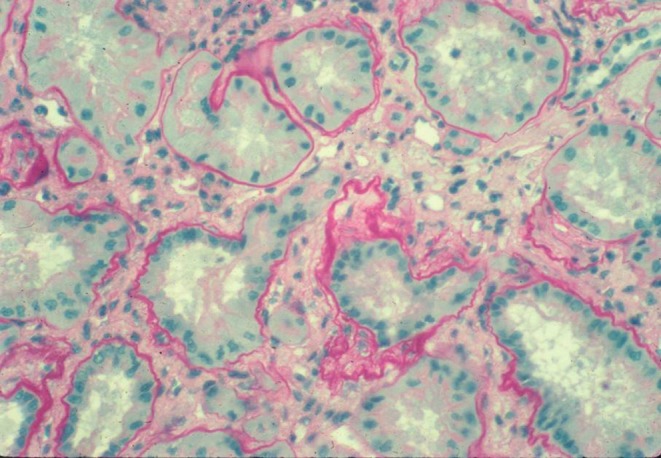
Renal histology of nephronophthisis. Cross-section of kidney showing diffuse interstitial fibrosis and various tubular changes. Some tubules are collapsed, others are surrounded by thickened tubular basement membranes. Note the laminated and wrinkled appearance of some tubular basement membrane segments as well as the abrupt attenuation of others in the same tubule (Light microscopy; magnification ×360; from Marie-Claire Gubler)

Renal ultrasound may be normal, with normal-sized kidneys, but renal parenchymal hyperechogenicity and loss of corticomedullary differentiation are often observed. At later stages, small cysts are present in the medulla [14, 15]. Renal biopsy shows severe tubular damage on light microscopy. Groups of atrophic tubules with thickened basement membranes alternate with groups of dilated or collapsed tubules. Homogeneous or multilayered thickening of tubular basement membranes is prominent, but disintegration of the basement membrane can also occur (Fig. 1). Abrupt transition from one abnormality to another is highly suggestive of juvenile NPH [16]. These various changes in the tubular basement membrane, although nonspecific, occur in NPH more extensively than in any kidney disorders with abnormal tubules. There is moderate to massive interstitial fibrosis with few inflammatory cells. The glomeruli are often normal, although secondary sclerosis is observed in advanced disease.

### Adolescent nephronophthisis

This form of autosomal recessive NPH has been called the adolescent form following the identification of the *NPHP3* gene in a large Venezuelan family in which ESRD occurred at a mean age of 19 years [17]. However, there is no clear correlation between the age at ESRD and the genotype, as some patients with an *NPHP3* mutation progress to ESRD before 10 years of age, whereas ESRD occurs in adulthood in some patients with *NPHP1* deletion. Histological lesions are similar to those observed in juvenile NPH.

### Infantile nephronophthisis

A chronic autosomal recessive tubulointerstitial nephritis with cortical microcyts progressing to ESRD before 2 years of age was initially described by Gagnadoux et al. [18]. The disease differs from juvenile NPH not only by its early onset but also by the histopathologic features. Whereas cystic dilatations of the collecting ducts are seen in these patients, the typical changes in the tubular basement membranes seen in juvenile NPH are usually absent. Ultrasonography usually shows moderately enlarged kidneys. Severe hypertension is common.

### Associated disorders

In 10–20% of cases, extrarenal symptoms are present, in particular, retinitis pigmentosa (RP) [Senior-Løken syndrome (SLS)], cerebellar ataxia [Joubert syndrome (JS)], oculomotor apraxia type Cogan, mental retardation, bone anomalies and hepatic fibrosis. Situs inversus and ventricular cardiac septal defect are associated in some patients with the infantile form (Table 1).

**Table 1:** Genetic heterogeneity and overlap of nephronophthisis (NPH), Senior-Løken, Joubert, and Meckel-Gruber syndromes

Locus	Chromosome	Gene*	Clinical manifestations
NPHP1/SLSN1	2q13	*NPHP1* (nephrocystin-1)	Juvenile nph (mild JBTS, mild RP, Cogan)
NPHP2	9q31	*NPHP2/INVS* (Inversin)	Infantile nph (RP, liver fibrosis, HT)
NPHP3/SLSN3	3q22	*NPHP3* (nephrocystin-3)	Juvenile nph (liver fibrosis, RP)
NPHP4/SLSN4	1p36	*NPHP4 *(nephrocystin-4 or nephroretinin)	Juvenile nph (Cogan, RP)
NPHP5/SLSN5	3q21	*NPHP5/IQCB1*	Juvenile nph + severe RP
NPHP6/SLSN6/JBTS5/MKS4	12q21	*NPHP6/CEP290*	Juvenile nph + JBTS + severe RP, isolated RP, (MKS)
NPHP7	16p	*NPHP7/GLIS2*	Juvenile nph
NPHP8/JBTS7/MKS5	16q	*NPHP8/RPGRIP1L*	Juvenile nph + JBTS (MKS)
NPHP9	17q11	*NPHP9/NEK8*	Juvenile and infantile nph

*JBTS* Joubert syndrome type B, *RP* retinitis pigmentosa, *MKS* Meckel-Gruber syndrome, *HT* arterial hypertension

*The name of the protein is indicated when it is not the same as the gene

**Fig. 2:**
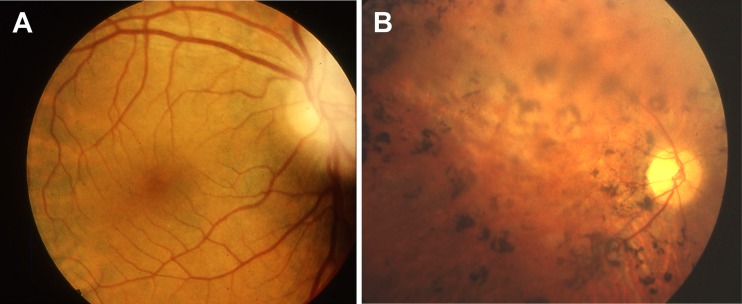
Retinitis pigmentosa. Ophtalmoscopic examinations of a control subject (**a**) and an affected individual (**b**) showing typical retinitis pigmentosa fundus characterized by very thin retinal vessels, retinal pigment epithelium atrophy, abnormal pigmentary migrations, and pallor of the optic disk

SLS, in which tapetoretinal degeneration (also known as RP) accompanies juvenile NPH is seen in approximately 10–15% percent of cases. Initially, Senior and Løken described patients with early and severe visual impairment resembling Leber congenital amaurosis [19, 20], but the syndrome has thereafter been extended to all patients with NPH and degenerative retinopathy. In the late-onset form, children may develop night blindness followed by complete visual impairment in the following years. Electroretinogram (ERG) shows complete extinction before RP may be observed by funduscopic examination (Fig. 2). Some patients have only an attenuated ERG, but visual acuity is normal. RP has been observed in association with mutations in most *NPHP* genes (except *NPHP7*), but whereas RP is always present and severe in patients with *NPHP5* and *NPHP6* mutations, the symptoms are in general mild in patients with mutations in the other *NPHP* genes. Georges et al. reported on four patients, from three different families, with RP responsible for severe visual impairment during childhood who developed chronic interstitial nephritis with histological lesions characteristic of NPH and renal failure only between 42 and 56 years of age [21]. No *NPHP1* deletion was found in these patients, but the other genes were not analyzed.

Nephrocystin proteins encoded by *NPHP4, 5, 6*, and *8* genes have been shown to localize to the connecting cilia between the inner and outer segments of the photoreceptors. All components necessary for assembly, maintenance, and turnover of the outer segment where the phototransduction takes place are synthesized in the cell body and are transported through the connecting cilia. Proteins implicated in other syndromes with retinal degeneration, such as Bardet-Biedl [22], Alstrom [23] or Usher [24] syndromes, have been shown to be localized to the connecting cilia where they are probably involved in the transport of phototransduction proteins, i.e. rhodopsin. Perturbation of these transporters leads to degeneration of the photoreceptor [25].

**Fig. 3:**
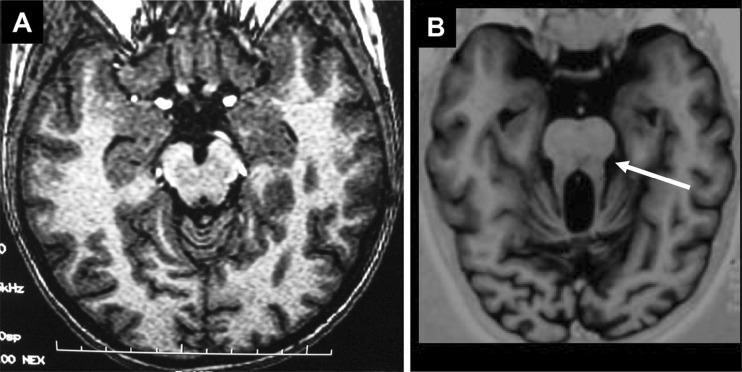
Molar tooth sign on brain magnetic resonance imaging (MRI). Brain MRI axial image at the level of the superior cerebellar peduncles of a control subject (**a**) and an affected individual (**b**) showing abnormally increased depth of the interpeduncular fossa, narrowing of the midbrain tegmentum, and thickening of the superior cerebellar peduncles, all of which contribute to the radiologic feature known as the molar tooth sign (*white arrow*)

JS is an autosomal recessive neurological disorder that associates congenital hypotonia evolving into cerebellar ataxia, developmental delay, oculomotor apraxia, and abnormal breathing pattern during the first month of life. JS is characterized by a complex cerebellar and brainstem malformation, the so-called “molar tooth sign” (MTS) observed by magnetic resonance imaging (MRI) (Fig. 3). JS can be associated with juvenile NPH and/or retinal involvement (JS type B). To date, three gene loci have been mapped to 9q34.3 (*JBTS1*), 11p11.2-q12.3 (*JBTS2*), and 6q23 (*JBTS3*), and mutations in the *AHI1* (Abelson helper integration site) gene have been identified in *JBTS3*-linked families with a pure cerebellar phenotype [26–28].

*NPHP1* homozygous deletions (identical to the deletions observed in patients with isolated NPH) have been identified in a small percentage of patients with a mild neurological form of JS and NPH (and RP in one case) [29, 30]. A recent survey of 56 families with *NPHP1* deletion revealed that 5 (8.9%) had JS-related disorders with a variable phenotype. Most of them had no mental retardation, the characteristic MTS aspect was present in only one family, and thickened superior peduncles was the only malformation on brain MRI in two other families, whereas it was unremarkable in one family [29]. Recently, mutations in the *NPHP6/CEP290* [31, 32] and *NPHP8/RPGRIP1L* [33, 34] genes have been found in patients with JS. They were associated with severe RP in the first case, whereas there was no or only mild retinopathy in patients with *RPGRIP1L* mutations. In a series of 28 patients with NPH and at least one JS-related neurological symptom, Tory et al. found *NPHP1* and *NPHP6* homozygous or compound heterozygous mutations in 13 (46%) [35].

Meckel-Gruber syndrome (MKS) is an autosomal recessive lethal disorder characterized by central nervous system malformation (typically occipital encephalocele), postaxial polydactyly, cystic kidney dysplasia, and ductal proliferation in the portal area of the liver [34, 36]. Interestingly, mutations in the *NPHP6, NPHP8*, and *MKS3* genes have been found in patients with MKS as well as in patients with JS, suggesting that these two conditions represent a broad spectrum of the same underlying disorder [34, 36, 37]. As with other genes implicated in cystic kidney diseases, most mutated proteins responsible for MKS and JS have been shown to be localized to kidney primary cilia, further suggesting a connection between these syndromes.

Bone anomalies can lead to phalangeal cone-shaped epiphyses, which are usually associated with other extrarenal manifestations (Saldino-Mainzer syndrome) [38]. Other skeletal dysplasia are associated in different syndromes. Hepatic involvement may be characterized by hepatosplenomegaly and portal fibrosis, with no or only mild bile duct proliferation [39–41]. Mutations of the *NPHP3* gene were reported in affected members with hepatic fibrosis and NPH from one family [17].

Situs inversus has been reported in a patient with infantile NPH and mutation of the *NPHP2* gene [42]. This patient also had a cardiac ventricular septal defect (Table 2).

**Table 2:** Extrarenal manifestations in nephronophthisis

Ocular	
Isolated oculomotor apraxia (Cogan syndrome)	
Retinitis pigmentosa (Senior-Løken syndrome)	
Coloboma	
Nystagmus (Joubert syndrome)	
Ptosis (Joubert syndrome)	
Neurological	
Mental retardation (Joubert syndrome or isolated)	
Cerebellar ataxia with vermis hypoplasia (Joubert syndrome)	
Hypopituitarism (RHYNS syndrome)	
Liver	
Elevation of hepatic enzymes	
Fibrosis, biliary duct proliferation (Boichis syndrome)	
Skeletal	
Phalangeal cone-shaped epiphyses (Saldino-Mainzer or cono-renal syndrome)	
Short ribs (Jeune or asphyxiating thoracic dystrophy syndrome)	
Postaxial polydactyly	
Skeletal dysplasia (Sensenbrenner syndrome or cranioectodermal dysplasia)	
Other:	
Situs inversus	
Cardiac malformations	
Bronchitis^a^	
Sterility^a^	
Hyperlipemia^a^	
Ectodermal dysplasia (Sensenbrenner syndrome)	

^a^Personal data

Several other syndromes that feature NPH have been described, such as Jeune [43], Ellis van Creveld (chondroectodermal dysplasia) [44], RHYNS (retinitis pigmentosa, hypopituitarism, and skeletal dysplasia) [45], Alstrom [46], Sensenbrenner (cranioectodermal dysplasia) [47, 48], and Arima-Dekaban [49] syndromes (Table 3). The description of these rare syndromes is beyond the scope of this paper.

**Table 3:** Syndromes featuring nephronophthisis or associated with mutations of *NPHP* genes

Senior-Løken	
Cogan	
Joubert (type B)	
Meckel-Gruber	
Saldino-Mainzer (cono-renal syndrome)	
Sensenbrenner (cranioectodermal dysplasia)	
Ellis van Creveld (ectodermal dysplasia)	
Jeune (asphyxiating thoracic dystrophy syndrome)	
RHYNS (retinitis pigmentosa, hypopituitarism, and skeletal dysplasia)	
Alstrom (retinal dystrophy, hearing impairment, obesity, type 2 diabetes mellitus)	
Arima-Dekaban	
Boichis	

## Genetics

**Fig. 4:**
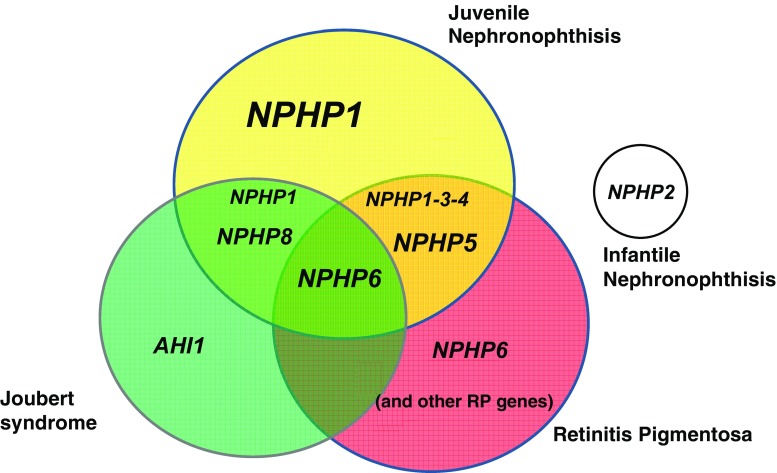
Genes implicated in nephronophthisis, retinitis, and Joubert syndrome. Different associations between these syndromes and the corresponding mutated genes. The most frequently mutated genes are indicated in *large characters*. *NPHP7* and *NPHP9* genes are not represented because of their low mutation rate. Extrarenal disorders associated with mutation in the *NPHP2* gene are not indicated. Two patients with *NPHP8* mutations and mild retinitis have been reported [34, 74]

Positional cloning and candidate-gene approaches led to the identification of causative genes. To date, mutations in eight different genes (*NPHP1, 3, 4, 5, 6, 7, 8,* and *9*) have been identified in juvenile NPH, whereas in the infantile form, mutations have been found in the *NPHP2* gene [8] (Fig. 4 and Table 2).

### *NPHP1* gene

**Fig. 5:**
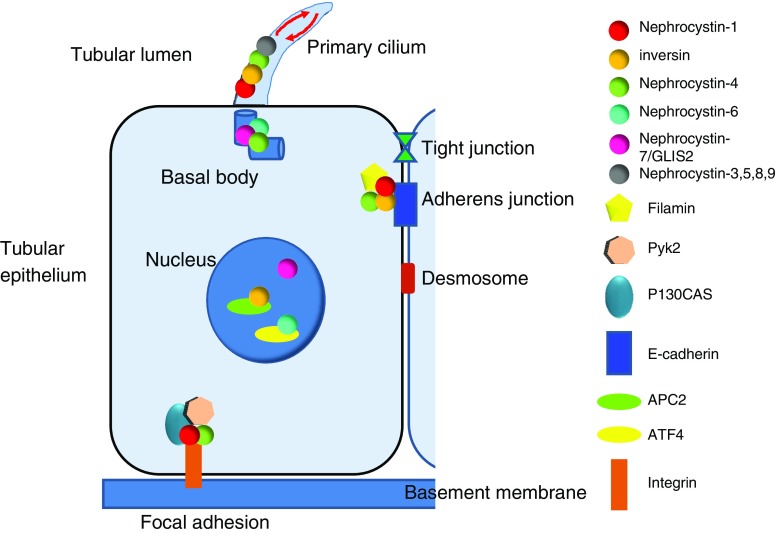
Schematic representation of the tubular epithelium and subcellular localization of the nephrocystin proteins. Nephrocystins localize to different subcellular compartments within the cell in a cell-cycle-dependent manner. Most nephrocystins interact with one another, forming a nephrocystin complex. In polarized renal tubular cells, all nephrocystin proteins localized to the primary cilia at the base of the cilium (basal body) and in a punctate pattern along the ciliary axoneme, suggesting their transport along the microtubule system (*red arrows*). Nephrocystin-1, nephrocystin-4, and inversin also localize to the cell–cell junctions and interact with focal adhesion proteins (p130Cas, Pyk2). Nephrocystins also associate with proteins associated directly with the microtubular and actin cytoskeleton (tubulins, tensin, and filamin). These localizations suggest a role for nephrocystins in modulating the cytoskeleton and maintaining epithelial-cell polarity. During cell cycle, nephrocystins localize to the centrosome. Moreover, during cell division, inversin, nephrocystin-4 and nephrocystin-6 localize to the mitotic spindle. In addition, inversin and nephrocystin-6 bind the anaphase-promoting complex (APC2) and activating transcription factor (ATF4), respectively, suggesting their potential involvement in cell-cycle regulation. The transcription factor nephrocystin-7/GLIS2 localized to both nucleus and primary cilia, as did the other NPHP proteins in renal epithelial cells

In 1993, the first gene responsible for juvenile NPH was localized on chromosome 2q13 by positional cloning in consanguineous families [50]. Homozygous deletions of about 250 kb in the 2q13 region were initially detected in 70% of patients [51, 52] and allowed identification of the responsible gene, *NPHP1*, in 1997. *NPHP1* contains 20 exons and encodes for a protein, named nephrocystin or nephrocystin-1, that has an src homology 3 (SH3) and coil-coiled domains that interact with proteins (including products of other NPHP genes) [53, 54]. Nephrocystin and its partners are localized at the cell–cell junction (adherens junction) and at the cell–matrix interface (focal adhesion), suggesting important functions in maintaining tubular epithelium (Fig. 5) [55, 56]. Moreover, nephrocystin-1 is also localized at the primary cilia-like proteins associated with other cystic kidney diseases, such as polycystins. The detection of homozygous mutations by polymerase chain reaction (PCR) amplification permits fast and accurate diagnosis of the disease without the need for renal biopsy. In large series of patients with a presumptive diagnosis of NPH based mainly on clinical and radiological data, *NPHP1* homozygous deletion is present in 20–40% of the cases ([8] and personal data). Heterozygous deletions are found in 6% of patients, with concomitant point mutation of the *NPHP1* gene on the second allele (personal data). Whereas most patients with *NPHP1* deletions or mutations have no extrarenal symptoms, a moderate form of retinal degeneration [57] or JS has been reported in some cases [29, 30, 35, 57].

### *NPHP2* gene

Mutations in the *NPHP2* (*INVS)* gene are responsible for the infantile form of NPH. This gene, located on chromosome 9q31, encodes inversin, a protein that is critical for normal left–right patterning in the vertebrate embryo [42, 58]. In proximal tubular cells, inversin is associated with nephrocystin-1 and with components of microtubule cytoskeleton [59]. It is localized to different subcellular compartments, including nuclei, cytoplasm, and cell–cell junction [60]. Its interaction with APC2 suggests that inversin might play a role during the cell cycle [61]. Knockout mice for the *INVS* gene show large renal cysts, altered left–right laterality (*situs inversus*), and hepatobiliary-duct malformations.

### *NPHP3* gene

*NPHP3* located on chromosome 3q22 was initially mapped in a large family of NPH patients from Venezuela [62]. Mutations in *NPHP3* were described in families with renal disease alone, as well as in families with renal disease associated with hepatic fibrosis or retinal degeneration [17]. Age at onset of ESRD was highly variable in patients with *NPHP3* mutations from 4 to 37 years of age. Recently, two NPH patients with ESRD at 4 years of age were found to carry *NPHP3* mutations [63]. In another recently published by Bergmann et al., *NPHP3* mutations were found in patients with a broad clinical spectrum of early embryonic patterning defects comprising situs inversus, polydactyly, central nervous system malformations, structural heart defects, preauricular fistulas and multicystic kidneys with perinatal death in some cases [64]. *NPHP3* encodes a 1,330 amino acid protein with a tubulin-tyrosine ligase domain that interacts with nephrocystin. Interestingly, the *pcy* mouse, a spontaneously occurring renal cystic disease model that closely resembles NPH, harbors a homozygous missense mutation in the mouse *NPHP3* ortholog (*Nphp3*) that most likely causes the kidney phenotype [17]. Recent observations that the *pcy*-associated renal cystic disease is amenable to treatment with a vasopressin-2 receptor antagonist [65] opens new perspectives for potential therapeutic strategies in NPH, for which no effective treatment is available.

### *NPHP4* gene

The *NPHP4* gene located on chromosome 1p36 encodes a 1,426 amino acid protein called nephrocystin-4/nephroretinin [66]. Nephrocystin-4 interacts with nephrocystin-1 and is probably involved in the same intracellular signaling pathway [66]. Interestingly, nephrocystin-4 is conserved in the nematode *Caenorhabditis elegans,* which exhibits male mating phenotype defect upon *Nphp4* knockdown, a phenotype also observed with orthologs of polycystic kidney disease genes. Direct sequencing of the *NPHP4* gene has been performed in 250 individuals, 190 with isolated NPH, 50 with RP and ten with oculomotor apraxia [67]. Twenty-three different *NPHP4* mutations were found in 26 (10%) unrelated patients (13 with isolated NPH, eight with RP, and two with oculomotor apraxia). Of note, nephrocystin-4 localizes to the connecting cilium of photoreceptor cells and interacts with *RPGRIP1* [RP guanosine riphosphatase (GTPase) regulator interacting protein 1], a component of cone and rod photoreceptors that is mutated in patients with Leber amaurosis [68].

### *NPHP5* gene

In contrast to the previous *NPHP* genes, mutations in the *IQCB1* gene, now referred to as *NPHP5*, were reported only in patients with NPH in combination with severe retinal degeneration and early blindness—SLS [25]. *NPHP5* mutations, involving both alleles in all cases, were found in 16 of 92 patients with early onset RP [25]. NPHP5 encodes an IQ-domain protein called IQCB1 or nephrocystin-5 that is expressed in connecting cilia of photoreceptors, where it is associated with calmodulin and retinitis pigmentosa GTPase regulator (RPGR). Nephrocystin-5 is also present in the primary cilia of renal epithelial cells [25].

### *NPHP6* gene

The *NPHP6* gene, also known as *CEP290*, encodes a centrosomal protein that activates ATF4, a transcription factor involved in the control of the cell cycle. Thirteen different mutations in the *NPHP6* gene were initially reported in 12 families with JS [31]. Most of the patients had congenital blindness or severe visual defect in the first years of life. In two families (patients aged 3, 9.5, 10, and 15 years), there was no renal disease. Interestingly, the *NPHP6* gene was thereafter reported to be mutated in more than 20% of patients with severe congenital blindness but no renal involvement (Leber congenital amaurosis) [69, 70]. A hypomorphic mutation (c.2991+1655A>G) that creates a strong splice-donor site and inserts a cryptic exon in the *CEP290* ribonucleic acid (RNA) was detected in 16 (21%) of 76 unrelated patients with blindness but without clinical signs of renal disease. Moreover, these patients had no neurological symptoms typical of JS and had normal cognitive function [69]. Another group has confirmed that mutations in the *NPHP6* gene is the most common cause of Leber amaurosis [70]. Given the broad spectrum of the phenotype associated with *NPHP6* mutations, Helou et al. screened this gene in 99 patients with cerebellar ataxia (JS), 75 patients with RP, and 21 patients with isolated NPH and found mutations in seven, two, and one case, respectively [71]. In four patients, only single heterozygous mutations were found, and in one of them, an additional heterozygous *NPHP4* missense mutation was present, arguing for a digenic inheritance. A genome-wide linkage scan in families with MKS led to the identification of *NPHP6* mutations in some patients [36].

### *NPHP7* gene

The *NPHP7* gene, also known as the *GLIS2* gene, contains six coding exons and encodes a Kruppel-like zinc-finger transcription factor, which has been found mutated in one consanguineous Oji-Cree Canadian family with isolated NPH in three children who developed ESRD by 8 years of age. This gene seems to be very rarely involved, as no other mutation was found in a cohort of 470 individuals with NPH-like phenotypes [72]. The kidneys of mice with a targeted disruption of the *Glis2* gene are atrophic, with fibrosis starting at 8 weeks of age. Apoptosis is increased in renal tubular cells, whereas cell proliferation is not. Interestingly, the genes promoting epithelial–mesenchymal transition and fibrosis are up-regulated in the absence of *Glis2*.

### *NPHP8* gene

Mutations in a novel gene on chromosome 16, *RPGRIP1L*, have been found in patients with JS and in fetuses with MKS [34]. Interestingly, Delous et al. reported that MKS fetuses carried two truncating mutations, whereas JS patients carried missense mutations and/or one truncating mutation, suggesting a genotype–phenotype correlation [34]. RPGRIP1L is a cytosolic protein that colocalizes at the basal bodies, centrosomes, and primary cilia in renal tubular cells with nephrocystin-4 and nephrocystin-6 [34]. Interestingly, missense mutations found in JS patients decrease the interaction of RPGRIP1L with nephrocystin-4 without affecting its localization, suggesting that a defect in this association may contribute to the phenotype [33, 34]. In the same lines, *NPHP4* missense mutations known to cause NPH with RP also disrupt this interaction [33]. Recently, it was shown that Rpgrip1l participates in sonic hedgehog (Shh) signaling and by this means plays a critical role in patterning of the developing neural tube and limb through the cilium [73]. The *RPGRIP1L* gene was thereafter analyzed in a cohort of 56 patients with JS. *RPGRIP1L* mutations were identified in five kindreds, including six individuals (8%). Of note, patients with *RPGRIP1L* mutations had normal retina, except two patients out of 12 with moderate visual impairment [34, 74]. Additional clinical symptoms were present in some patients with *RPGRIP1L* mutations, such as liver fibrosis, postaxial polydactyly, pituitary agenesis, and partial growth hormone deficiency. These last findings indicate a possible overlap with RHYNS syndrome [34, 74].

### *NPHP9*

The jck cystic kidney mouse model is associated with a mutation in the *Nek8* gene [75]. Analysis of the *NEK8* gene in a cohort of 588 patients with NPH led to the identification of three missense mutations in patients with isolated NPH [76]. In one patient, an additional mutation in the *NPHP5* gene was also present. Interestingly, mutant forms of *NEK8* showed defects in ciliary localization.

### Genetic heterogeneity and oligogenism

Overall, *NPHP1* to *NPHP9* mutations have been reported in cases of juvenile NPH with or without extrarenal symptoms, except for mutations in *NPHP2* that have been found only in patients with infantile NPH. *NPHP1* mutations were found in ~20% to 40% of cases [8], whereas mutations in the other genes seem to account for a very low percentage of cases (Table 1). Analysis of the *NPHP1*, *NPHP3*, and *NPHP4* genes in a cohort of 94 different families have shown that a mutation or a deletion in one of these three genes was identified in 44 (47%) patients [77]. Interestingly, in six families, three mutations in two *NPHP* genes were found, whereas two mutations in two *NPHP* genes (*NPHP3* and *NPHP4*) were found in another kindred. Finally, a single mutation in one of these three genes was discovered in nine other patients [77]. In the same lines, Tory et al. found that some patients with NPH and at least one JS-related neurological symptom had both an *NPHP1* deletion and either a heterozygous *NPHP6* or *AHI1* mutation [35]. These findings demonstrate that similar to the inheritance patterns described in Bardet-Biedl syndrome (BBS), NPH, at least in some patients, follows a digenic or oligogenic inheritance with heterozygous mutations in different genes in the same patients. Sequencing of all the known NPH genes in a large cohort of patients will be necessary to validate this model and appreciate the mutation load that could account for the severity of the nephropathy as well as the extrarenal symptoms.

### The cilia connection

Cilia are present in almost all cells in the organism and act as sensory organelles that connect visual, mechanosensory, odorant or other stimuli to cell-cycle, control of epithelial architecture, or other yet unknown processes. The finding that polycystin-1 and polycystin-2—the proteins responsible for autosomal dominant polycystic kidney disease [78] and proteins involved in other cystic kidney diseases (BBS [22], oro-facio-digital syndrome [79])—were present in cilia in renal tubular cells indicates a possible connection between this organelle and cyst formation. It has been suggested that the primary cilium senses fluid movement in the renal tubules. The presence of cystoproteins (such as nephrocystins) in cilia of various organs (photoreceptor, ependymal cells, cholangiocytes, chondrocytes) explains the multiple organ involvement in some patients with NPH. To illustrate this unifying theory, the term ciliopathies has been coined to design all the syndromes related to dysfunction of ciliary proteins. For more information on this subject, the readers are referred to an excellent recent review [80].

## Conclusions

**Fig. 6:**
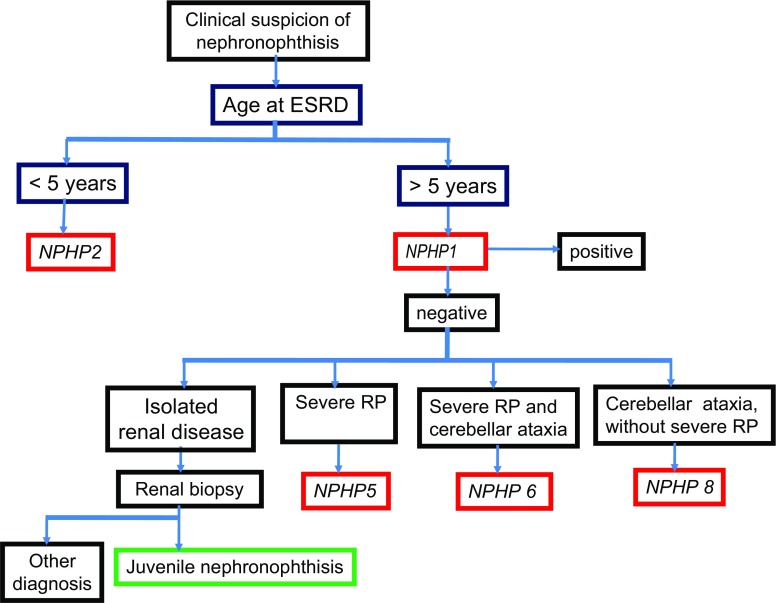
Decision algorithm for genetic analyses when nephronophthisis is suspected on clinical and radiological basis. At first, *NPHP1* or *NPHP2* genes should be screened for mutations, depending on the age at onset of end-stage renal disease. The other genes are analyzed in function of extrarenal symptoms. *NPHP3, NPHP4, NPHP7*, and *NPHP9* genes are currently not sequenced for diagnostic purposes because of their low mutation rate. *RP* retinitis pigmentosa

In conclusion, and from a practical point of view, the diagnosis of NPH should be considered if a child presents with polyuria, urinary sodium loss, growth failure, renal insufficiency without hematuria or proteinuria, normal blood pressure, and normal-sized kidneys without dilatation of the urinary tract. These patients should be screened for homozygous or heterozygous *NPHP1* deletion, which is found in 20–40% of cases. In the absence of such deletion, renal biopsy may be proposed to confirm the diagnosis. At present, screening for mutation in all the other NPHP genes is not routinely performed due to the low frequency of detected mutations and the high cost of the procedure. Patients with associated disorders should be considered separately. In case of severe RP, the *NPHP5* gene may be screened for mutations, whereas in patients with neurological symptoms such as cerebellar ataxia, the *NPHP6* and *NPHP8* genes should be analyzed first. In case of early onset tubulointerstitial nephritis with cortical microcysts, the *NPHP2* gene should be screened for mutation (Fig. 6).

## Appendix

### **Questions**

(Answers appear after the reference list)

1. The diagnosis of nephronophthisis may be suspected in a child with chronic renal failure and
  A) a past history of polyuria
  B) de Toni-Debré-Fanconi syndrome
  C) a chronic tubulointerstitial nephritis on renal biopsy
  D) two large kidneys on renal ultrasound
  E) consanguineous parents
2. The following extra-renal symptoms may be observed in a child with nephronophthisis
  A) hepatomegaly
  B) tapetoretinal degeneration
  C) cone-shaped epiphysis
  D) cerebellar ataxia
  E) diabetes mellitus
3. Children with mutations in the *NPHP2* gene
  A) develop end stage renal disease after 10 years of age
  B) have cortical microcysts on renal biopsy
  C) may present with situs inversus
  D) often develop severe hypertension
  E) may present with Senior-Løken syndrome
4. Kidneys in juvenile nephronophthisis are characterized by
  A) tubulo-interstitial fibrosis
  B) mesangial hypercellularity
  C) tubular basement membrane irregularities
  D) cortical cysts
  E) tubular atrophy
5. Juvenile nephronophthisis is genetically characterized by
  A) a recessive mode of inheritance
  B) mutations in *NPHP5* and *NPHP6* genes in patients with severe retinal dystrophy
  C) mutation in more than one gene in some patients
  D) a more severe disease in males
  E) *NPHP1* gene deletion in 20 to 30% of the patients
